# Risk factors for MERS coronavirus infection in dromedary camels in Burkina Faso, Ethiopia, and Morocco, 2015

**DOI:** 10.2807/1560-7917.ES.2017.22.13.30498

**Published:** 2017-03-30

**Authors:** Eve Miguel, Véronique Chevalier, Gelagay Ayelet, Med Nadir Ben Bencheikh, Hiver Boussini, Daniel KW Chu, Ikhlass El Berbri, Ouaffa Fassi-Fihri, Bernard Faye, Getnet Fekadu, Vladimir Grosbois, Bryan CY Ng, Ranawaka APM Perera, TY So, Amadou Traore, François Roger, Malik Peiris

**Affiliations:** 1 Cirad UPR AGIRs, Montpellier, France; 2 UMR CNRS, IRD, UM, 5290 MIVEGEC, Montpellier, France; 3 National Veterinary Institute, Addis Abeba, Ethiopia; 4 Institut Agronomique Vétérinaire Hassan 2, Rabat, Morocco; 5 INERA-CNRST, Ouagadougou, Burkina Faso; 6 School of Public Health, The University of Hong Kong, Hong Kong Special Adminstrative Region, China; 7 Cirad UMR SELMET, Montpellier, France; 8 Haramaya university, Dire Dawa, Ethiopia

**Keywords:** Density, herd size, milking activities, nomadic, sedentary, West - East gradient

## Abstract

Understanding Middle East respiratory syndrome coronavirus (MERS-CoV) transmission in dromedary camels is important, as they consitute a source of zoonotic infection to humans. To identify risk factors for MERS-CoV infection in camels bred in diverse conditions in Burkina Faso, Ethiopia and Morocco, blood samples and nasal swabs were sampled in February–March 2015. A relatively high MERS-CoV RNA rate was detected in Ethiopia (up to 15.7%; 95% confidence interval (CI): 8.2–28.0), followed by Burkina Faso (up to 12.2%; 95% CI: 7–20.4) and Morocco (up to 7.6%; 95% CI: 1.9–26.1). The RNA detection rate was higher in camels bred for milk or meat than in camels for transport (p = 0.01) as well as in younger camels (p = 0.06). High seropositivity rates (up to 100%; 95% CI: 100–100 and 99.4%; 95% CI: 95.4–99.9) were found in Morocco and Ethiopia, followed by Burkina Faso (up to 84.6%; 95% CI: 77.2–89.9). Seropositivity rates were higher in large/medium herds (≥51 camels) than small herds (p = 0.061), in camels raised for meat or milk than for transport (p = 0.01), and in nomadic or sedentary herds than in herds with a mix of these lifestyles (p < 0.005).

## Introduction

In September 2012, a novel coronavirus, Middle East respiratory syndrome coronavirus (MERS-CoV), was identified from a patient with a fatal viral pneumonia in Saudi Arabia. This coronavirus is genetically related, but not identical, to the severe acute respiratory syndrome (SARS) coronavirus which emerged in southern China in 2002 [1]. As of 21 March 2017, 1,917 human cases have been reported to the World Health Organization (WHO) with at least 684 deaths [2]. Most zoonotic infections have occurred in the Arabian Peninsula, particularly in Saudi Arabia, although nosocomial outbreaks arising from travellers coming from the Arabian Peninsula have been reported in Africa, Asia, Europe and North America. For example, between May and June 2015, 186 human infections in South Korea arose from one returning traveller [3], highlighting the cause for global public health concern.

Human disease ranges from mild or asymptomatic infection to a fulminant viral pneumonia progressing to severe respiratory failure and death. Dromedary camels are strongly suspected to be the source of human infections [4]. It is believed that humans can get infected via direct contact with mucous membranes of infected camels [5,6] or by consuming unpasteurised camel milk [7]. However, the virus has not been detected in camel urine [8] or in raw camel meat [9]. Secondary infections in humans are reported, especially within nosocomial settings [10,11] or to a smaller extent, within households [12], suggesting that human-to-human transmission may become efficient enough to trigger outbreaks beyond the current epicentre in the Middle East. The WHO has identified MERS-CoV as one of the pathogens of greatest concern for global public health for which few or no medical countermeasures exist [13]. To date there are no vaccines or antivirals available for MERS-CoV in humans [14]. Camel vaccines have given promising results with the use of a vaccinia Ankara (MVA) vectored vaccine [15].

MERS-CoV only causes mild respiratory symptoms in camels and it is consequently not easily recognised and difficult to diagnose clinically. High levels of seropositivity and virus detection rates have been observed in dromedary camels in the Arabian Peninsula [16,17]. MERS coronaviruses detected in camels are genetically very similar or identical to those infecting humans [18]. MERS-CoV antibodies have also been detected in dromedary camel populations of many countries outside the Arabian Peninsula. Serological studies in Africa indicate high seropositivity rates and the testing of retrospectively collected serum samples provide evidence that this virus has been infecting camels in East Africa since as early as 1983 [19]. More recent specimens collected between 2009 and 2013 show high rates of detection of MERS-CoV antibodies in camels in Egypt, Ethiopia, Kenya, Nigeria, Sudan and Tunisia and also in the Canary Islands [20,21].

Surprisingly, the only indication of locally acquired primary zoonotic human infections outside the Arabian Peninsula is the recent detection of antibodies against MERS-CoV in autochthonous livestock handlers in Kenya between 2013 and 2014 [22]. Possible reasons for the absence of reports of MERS-CoV infections in humans in Africa may include (i) underdiagnosis in humans due to a possible lack of awareness, lack of viral diagnostic capacity and weak healthcare systems, (ii) differences in virus strains or in camel breeds resulting in low infectiousness towards humans, (iii) differences in cultural practices in interaction between humans and dromedary camels, or any combination of these. Research recommendations from workshops on MERS-CoV in Doha April and Cairo May 2015, organised by the Food and Agriculture Organisation (FAO), the Organisation of the United Nations for Animal Health (OIE) and the WHO identified the apparent absence of human MERS-CoV infections in Africa despite intense virus circulation among dromedaries as a key research question [23]. In order to address this question, it is important to understand the ecological and farming husbandry factors that may promote the likelihood of MERS-CoV infection in camels in Africa.

We report a descriptive serological and virological survey of MERS-CoV from west to east across the African continent, which was conducted by sampling camels in Burkina Faso, Ethiopia and Moroco. Sampling was designed so as to also assess the influence of the herd size, camel function (raised for milk, meat or transport) and lifestyle (either nomadic, sedentary or a mix of the two lifestyles) on likelihood of MERS-CoV infection.

## Methods

### Study sites and camel farming

Nomadic, sedentary, mixed lifestyles and extensive, semi-extensive and intensive camel breeding systems occur in African ecosystems. Extensive system/nomadic lifestyle are characterised by the use of natural resources, low inputs, and herd mobility [24]. However, camel husbandry practices and the use of camels have changed in the last five decades in the following ways: (i) increasing camel populations in settled livestock farming systems, (ii) use of camels in agriculture-related work, (iii) camel trade being more closely market integrated and (iv) increasing importance of camels for the sustainability and resilience of farms which traditionally relied on cattle [25]. These changing camel herding practices lead to sedentary or mixed lifestyles with intensive or semi-intensive camel production systems (milk, meat, skin etc.) [26]. Usually, camel calves are suckled by their mother during the first year of life. Camels are considered as young and sexually immature until 2–4 years-old. Males represent 20 to 40% of the herd [27]. Adult males are separated from females and young camels in non-extensive systems because of their aggressiveness associated with sexual behaviour. In extensive systems, the male is let with non-lactating females for reproduction only and during the rutting season. The contacts of adult males with young (less than 4 years-old) camels is not common.

**Figure 1:**
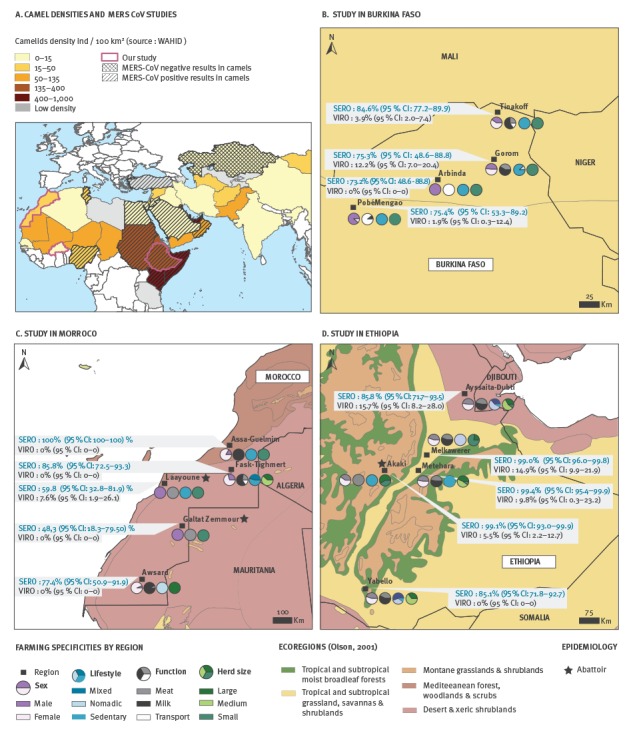
A. Camel densities in Africa, Middle East, and Asia with areas with prior serological evidence for MERS-CoV infection in camels, and B–D. sampling sites of this study, with serological and virological MERS-CoV detection rates in Burkina Faso, Ethiopia and Morocco, February–March 2015

Camel density increases from North to East Africa through the Sahelian strip with the highest densities recorded in the Greater Horn of Africa which harbours 60% of the world population [9] (i.e. with 400–1,000 individuals/100 km^2^ in Kenya and Somalia for instance; Figure 1A). Our sampling design covers Burkina Faso, Ethiopia and Morocco and a diversity of farming systems in different contexts (Figure 1B–D). Camel density is estimated at 0.07 individuals/km^2^ with 18,374 camels in Bukina Faso, 1.99 individuals/km^2^ with 2,245,581 camels in Ethiopia, and 0.44 individuals/km^2^ with 197,550 camels in Morocco [27]. Camel population densities are available at the country level only. However as camels are dependant on specific ecosystems which are mainly deserts or tropical and subtropical grasslands, savannas and shrublands [28] (Figure 1B–D), they are not distributed homogeneously in each country. Unfortunately, statistics on regional densities are not available.

Ind: individuals; MERS-CoV: Middle East respiratory syndrome coronavirus; SERO: serological detection rate; VIRO: virological RNA detection rate.

A. Update of the MERS-CoV negative and positive serological results in camels published in literature (hatched areas) and the countries selected for the study i.e. Burkina Faso, Ethiopia and Morocco (with thick borders).

B, C, D. Regions sampled by country with their farming specificities (different pie charts according to camel sex, function, lifestyle and herd size), the local ecoregions, and the estimated seropositivity and viral RNA detection rates by region from the cross-sectional survey done in February–March 2015.

### Field work

The field work was done between February and March 2015 in collaboration with the animal health institutes from Burkina Faso (Laboratoire de Biologie et Santé Animales - INERA-CNRST), Ethiopia (National Veterinary Institute) and Morocco (Institut Agronomique et Vétérinaire Hassan II). Cross-sectional studies were carried out simultaneously in the three countries. Blood (for serological analyses) and nasal swabs (for virological analyses) were collected from camels. The swabs were placed in virus medium transport. The blood samples were allowed to clot at room temperature and the serum extracted with a pipette. Swabs and sera were placed in cool box with ice packs if a −80 °C freezer was reachable in 48 h or otherwise frozen in a liquid nitrogen tank. On arrival at the national laboratory, all the samples were stored in a −80 °C freezer before their shipment to the international reference laboratory at the University of Hong Kong, for MERS-CoV serological and virological analyses. Questionnaires to ascertain camel habitats, environment and farming practices were administered to the farmers by veterinarians after specimen collection.

Camels raised for three distinct functions (milk, meat or transport) were sampled. Herd size was classified into three categories (small with ≤ 50 camels, medium with 51 to 150 camels and large with 151 to 300 camels). Samples were collected at two types of sites, with the majority taken at farms (1,301 samples from 80 herds) and some at abattoirs (199 samples from 6 herds) in Ethiopia and Morocco (Table 1).

**Table 1:** Location, number^a^ and characteristics of camels sampled for a cross-sectional serological and virological surey on MERS-CoV, Burkina Faso, Ethiopia and Morocco February–March 2015 (n=1,500 camels)

Country	Region	Tot Inds.	Inds./herd	Sex		Function			Herd size^b^			Lifestyle			Type	
				Female	Male	Meat	Milk	Transport	Large	Medium	Small	Mixed	Nomadic	Sedentary	Abattoir	Farm
**Burkina Faso**	Gorom	127	12	66	61	52	74	1	0	0	127	0	16	111	0	127
	Tinakoff	289	10	172	117	73	171	45	0	0	289	0	0	289	0	289
	Arbinda	47	24	0	47	0	0	47	0	0	47	0	0	47	0	47
	PobéMengao	62	31	7	55	0	7	55	0	0	62	0	0	62	0	62
**Ethiopia**	Akaki^c^	100	25	60	40	100	0	0	59	0	41	0	0	100	100	0
	Ayssaita-Dubti	99	20	52	47	46	53	0	33	56	10	36	56	7	0	99
	Melkawerer	199	22	111	88	88	111	0	45	0	154	0	199	0	0	199
	Metehara	140	23	74	66	66	74	0	61	65	14	0	0	140	0	140
	Yabello	94	24	52	42	40	54	0	33	61	0	52	33	9	0	94
**Morocco**	Assa-Guelmim	24	12	20	4	0	4	0	0	0	24	0	0	24	0	24
	Awsard	66	66	62	4	5	60	1	66	0	0	0	66	0	0	66
	Fask-Tighmert	154	15	109	45	35	95	24	59	95	0	71	0	83	0	154
	Galtat Zemmour^c^	16	16	0	16	16	0	0	0	0	16	0	0	0	16	0
	Laayoune^c^	83	83	0	83	83	0	0	0	0	83	0	0	30	83	0

Inds: individuals; MERS-CoV: Middle East respiratory syndrome coronavirus.

^a^ Number of camels sampled according to variables tested in the modelling of spatial variations and risk factors for estimating the propability of detecting MERS-CoV antibodies and RNA.

^b ^A small herd comprised ≤ 50 camels, a medium herd between 51 and 150 camels and a large herd between 151 and 300 camels.

^c^ Sampling took place at an abattoir.

Sampled camels were classified into one of three distinct lifestyles (nomadic, sedentary or a mix of nomadic and sedentary). The mixed lifestyle is characterised by a seasonal spatial movement of less than 100 km for accessing new ressources while the nomadic lifestlyle was defined as travelling throughout the year over distances up to hundreds of kilometers.

Each region has specifities in term of farming practices (Figure 1B–D). For example in Morocco, camels bred for meat are mainly young males in small herds and are sent to the abattoir (i.e. Laayoun) while camels bred for milk are females living in large nomadic herds (i.e. Awsard) (see Figure 1C for the specificities by region covered in the study).

### Biological analyses

Specimens were shipped on dry ice to the University of Hong Kong. Serum samples were tested for MERS-CoV antibodies at a screening dilution of 1:20 using an extensively validated MERS-CoV (strain EMC) spike pseudoparticle neutralisation test [29]. Selected positive sera were confirmed using microneutralisation tests in biosafety level (BSL)3 containment [30]. Total nucleic acid was extracted from swab samples using the EasyMag (Biomerieux) system and tested for the presence of MERS-CoV RNA using the upstream of the envelope gene (UpE) reverse transcription-quantitative PCR (RT-qPCR) hydrolysis probe assay. All positive specimens were confirmed by a second RT-qPCR assay targeting the open reading frame (ORF) 1a region of the genome as previously described [18].

### Statistical models for depicting serological and virological status according to geography and risk factors

Generalised linear mixed models (GLMM), with binomial error structures, were used to depict variations in serological and virological status according to individual characteristics (sex and age), spatial localisation (country and regions) and farming practices (camel’s function, herd size, sampling place and lifestyle). The results from the abattoirs were not included in the risk factor modelling due to the difficulty to get reliable information on the farms where the animals were raised. However, data from the abattoir were included for the statistical modelling of geographical variations of serological and virological rates. Indeed, the presence of an abattoir in a region may strongly influence the likelihood of infection in that region. In the statistical models, the dependent variable was binary: the serological and virological status of an individual was designated either positive or negative according to the result of the tests presented above. Because individuals were aggregated in herds, independence of statistical units was questionable. Herd random effects were thus included in the models. Goodness of fit was assessed through the Pearson overdispersion test [31]. Selection among models including different combinations of the explanatory variables was performed using Akaike information criterion [31,32].

As some of explanatory variables may be collinear, two-by-two comparisons of explanatory variables were used to assess possible confounding influences. Cramer’s V (CrV) test was used for categorical variables and R^2^ obtained from linear models for continuous variable. When the statistic is close to 1 for R^2^, or larger than 0.4 for CrV test, the two explanatory variables are considered as collinear and were not be used in the same statistical models. All the statistical analyses were performed using the software R [33].

## Results

In total 1,500 camels were sampled, between February and March 2015, from 86 herds (Figure 1B–D and Table 1). This included 525 camels in Burkina Faso from 43 herds from four regions (Tinakoff, Gorom, Arbinda, PobéMengao); 632 camels in Ethiopia from 28 herds from five regions (Ayssaita-Dubti; Melkawerer; Akaki-Addis Abeba; Metehara; Yabello) and 343 camels in Morocco from 15 herds from five regions (Assa-Guelmim, Fask-Tighmert, Laayoune, Galtat Zemmour, Awsard).

### Collinearity tests

Camel’s function and sex were strongly associated with each other (Table 2 and Figure 1B–D) (CrV = 0.86), as were region and lifestyle (CrV = 0.78); herd category and region (CrV = 0.70); region and function (CrV = 0.61); herd category and country (CrV = 0.50); function and lifestyle (CrV = 0.41). The strongest association was between region and type of specimens (i.e. farm or abattoir) with a Cramer’s V equal to 1. Region and age were also slightly collinear with a R^2^ of 0.20.

**Table 2:** Colinearity index among variables explaining MERS-CoV seropositivity and viral RNA detection rates

Colinearity index	Age	Sex	Function**^a^**	Region	Lifestyle**^b^**	Type**^c^**	Herd category**^d^**	Country
**Age**	1.00							
**Sex**	0.03	1.00						
**Function^a^**	0.04	**0.86**	1.00					
**Region**	0.20	0.38	**0.61**	1.00				
**Lifestyle^b^**	0.01	0.12	0.17	**0.78**	1.00			
**Type^c^**	0.01	0.05	**0.41**	**1.00**	0.25	1.00		
**Herd category^d^**	0.03	0.22	0.18	**0.70**	0.40	0.19	1.00	
**Country**	0.13	0.14	0.31	**1.00**	**0.41**	0.25	**0.50**	1.00

Two-by-two comparisons of explanatory variables were used to assess possible confounding influences. Cramer’s V test was used for categorical variables and R^2^ obtained from linear models for continuous variables. When the statistic is close to 1 for R² or larger than 0.4 for CrV test, the two explanatory variables are considered as collinear and cannot be used in the same statistical models. When the statistic is larger than 0.4 for the CrV test the result is in bold.

^a ^The function refers to whether the camel was bred for milk, meat or transport.

^b^ The lifestyle refers to whether the camel was sendentary, nomadic or had a mix of sedentary and nomadic lifestyles.

^c^ The type refers to whether samples were taken, such as a farm or a slaughterhouse.

^d^ The herd category refers to the herd size (small with ≤ 50 camels, medium with 51 to 150 camels and large with 151 to 300 camels).

### Modelling spatial variations

At the country scale, seropositivity and virus detection rates varied significantly across regions with p-values < 0.005 for the regional effect (i.e. seropositivity and virus detection rates) (Table 3 and Figures 1 B–D).

**Table 3:** Multivariate modelling used to depict variations in serological and virological status according to individual characteristics (sex and age), spatial localisation (country and regions) and farming practices (camel’s function, herd category, and lifestyle) using data from Morocco, Burkina Faso and Ethiopia, February–March 2015

GLOBAL MODEL Multivariate models herd as random effect (1|herd)			
**VARIABLES**	**AIC**	**Variables**	**P value**
**SEROLOGY**			
**Spatial variations**			
Age + country + sex + (1|herd)	1,047.8	NA	NA
Age + region + sex + (1|herd)	1,029.9	Age	0.001
		Region	< 0.005
		Sex	0.068
**Farming risk factors**			
Age + sex + lifestyle^a^ + herd category^b^ + (1|herd)	960.9	NA	NA
Age + sex + type^c^ + lifestyle^a^ + herd category^b^ + (1|herd)	960.4	NA	NA
Age + function^d^ + lifestyle^a^ + herd category^b^ + (1|herd)	961.4	Age	0.032
		Function^d^	0.016
		Lifestyle^a^	< 0.005
		Herd category^b^	0.061
**VIRUS DETECTION RATE**			
**Spatial variations**			
Age + country + sex + (1|herd)	640.5	NA	NA
Age + region + sex + (1|herd)	619.7	NA	NA
Age + region + (1|herd)	618.8	Age	0.369
		Region	< 0.005
**Farming risk factors**			
Age + function^d^ + lifestyle^a^ + herd category^b^ + (1|herd)	651.6	NA	NA
Age + function^d^ + lifestyle^a^ + (1|herd)	647.8	NA	NA
Age + sex + (1|herd)	651.7	NA	NA
Age + function^d^ + (1|herd)	646.1	Age	0.067
		Function^d^	0.015

AIC: Akaike information criterion.

AIC Selection and p values. Each model depicts the variation of serological and virological status (response variable: positive/negative results) according to explanatory variables (age, country, region, sex, lifestyle, herd category, camel function). Herd random effects are included in the models (1 |herd). Selection among models including different combinations of these explanatory variables was performed using AIC where a difference of 2 is required for selecting a model which combined variables influencing significantly the response variable.

^a ^The lifestyle refers to whether the camel was sendentary, nomadic or both.

^b ^Herd category refers to the size of the herd (small with ≤ 50 camels, medium with 51 to 150 camels and large with 151 to 300 camels).

^c ^The type refers to whether samples were taken such as a farm or a slaughterhouse.

^d^ The function refers to whether the camel was bred for milk, meat or transport.

In different regions of Burkina Faso, seropositivity rates ranged from 73.2% (95% confidence interval (CI): 48.6–88.8) to 84.6% (95% CI: 77.2–89.9) and virus detection from 0% (95% CI: 0–0) to 12.2% (95% CI: 7–20.4) (Figure 1B). In Ethiopia seropositivity rates ranged from 85.1% (95% CI: 71.8–92.7) to 99.4% (95% CI: 95.4–99.9) and the viral RNA detection rates from 0% (95% CI: 0–0) to 15.7% (95% CI: 8.2–28.0) (Figure 1D). In Morocco, seropositivity rates ranged from 48.3% (95% CI: 18.3–79.5) to 100% (95% CI: 100–100) and viral RNA detection rates from 0% (95% CI: 0–0) to 7.6% (95% CI: 1.9–26.1) (Figure 1C).

Taking the countries globally (irrespective of regional variation), seropositivity and viral RNA detection rates were higher in Ethiopia, as compared with Burkina Faso and Morocco.

### Modelling risk factors

**Figure 2:**
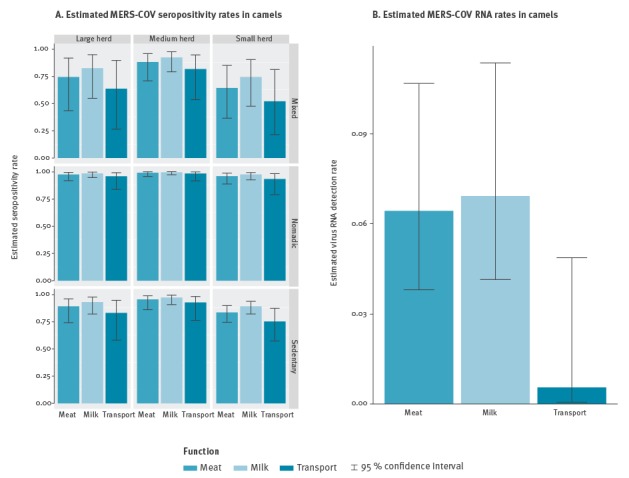
MERS-CoV seropositivity and viral RNA detection rates estimated by modelling according to significant risk factors, Burkina Faso, Ethiopia and Morocco, February–March 2015

In the modelling of variations of seropositivity rates (Figure 2A and Table 3), the retained explanatory variables were herd size category (p-value = 0.061), camel’s function (p-value = 0.01) and lifestyle (p-value < 0.005).

MERS-CoV: Middle East respiratory syndrome coronavirus.

**Figure 3:**
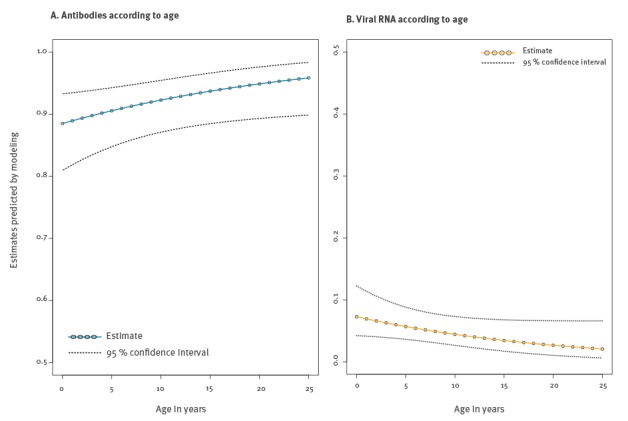
MERS-CoV seropositivity (antibodies) and viral RNA detection rates in camels estimated by modelling according to age, Burkina Faso, Ethiopia, Morocco, February–March 2015

Higher seropositivity rates were observed (i) in large/medium herds as compared with small herds; (ii) in camels bred for meat or milk as compared with camels bred for transport, and (iii) in nomadic or sedentary herds than in herds with a mix of these lifestyles. Seropositivity rates also increased with age (p-value = 0.032; Figure 3) and were higher in females than in males.

MERS-CoV: Middle East respiratory syndrome coronavirus.

In the modelling of virus RNA detection rate (Figure 2B and Table 3), camel’s function had a significant effect (p-value = 0.01) with higher viral RNA detection rates observed in camels bred for milk or for meat as compared with transport. Probability of detecting virus RNA also decreased with increasing age (p-value = 0.06; Figure 3) and was higher in females than in males (according to collinearity index as the variables function and sex strongly associated).

## Discussion

Our results support the contention that the MERS-CoV is actively circulating in camel populations in Burkina Faso, Ethiopia, Morocco and likely across all North, West and East Africa. The finding of high levels of seropositivity rates, which is an indication of infection at some point in the animals’ life time, was not surprising, and was in keeping with data from previous studies in Ethiopia and in other parts of Africa [19,20,34]. This study, however, presents the first evidence of MERS-CoV activity in Burkina Faso and in Morocco (Figure 1A and previous mapping of MERS serological studies). There are few reports of virus detection in camels in Africa. Here, MERS-CoV RNA was detected at a relatively high rate of up to 15.7% (95% CI: 8.2–28.0) in Ethiopia, followed by Burkina Faso with up to 12.2% (95% CI: 7–20.4) and Morocco up to 7.6% (95% CI: 1.9–26.1).

There is an apparent gradient of virus RNA positivity adjusted for age (Table 3) from west to east which could be explained by a gradient in camel density (Figure 1A), in addition to other drivers such as climate, migratory roads and national and international camel exchanges. Since Ethiopia is a main exporter to the Arabian Peninsula through two main ports in Djibouti and Somalia [35], the virus transmission dynamics in this region is of particular interest.

We observed an increase in seropositivity rate with age which confirms the trend observed in Ethiopia in a previous study [20]. We found a higher virus RNA detection rate in young animals compared with older animals which could be related to a lack of prior immunity as published in previous studies in Saudi Arabia [36]. Young animals were naïve and more susceptible to virus infection (Figure 3) [37].

The role of camel density in shaping the large spatial scale (i.e. national) variation pattern in seropositivity and virus RNA detection rates is supported by the identification, at fine scale (i.e. herd), of a herd size effect on serological prevalence. Higher seropositivity rate was found in large or medium size herds as compared with small herds, suggesting that the transmission of the virus is density dependent. More studies are now necessary to better describe the virus transmission dynamics within herds and between herds, with mechanistic models accounting for a disease transmitted through close contact and the possibility of reinfections [38]. Such a model would allow to determine the minimum size of a camel herd required for the MERS-CoV to persist in that herd without ‘fadeouts’: i.e. critical community size [39].

Another point highlighted by our study as a risk factor is the function of camels which is also related to sex. Camels raised for milking (which are females) show the highest serological prevalence followed by camels raised for their meat (which are mostly males) and lastly, camels used for transport activities (which are also mostly males), which have the lowest seroprevalence (Table 3 and model selection). The higher seropositivity rate in females bred for milking could be related to the high viral RNA detection rates in younger animals, e.g. calves [37]. A plausible hypothesis could indeed be that young camels who lack antibodies have a high probability of being infected and in turn expose the mothers to infection or reinfection. The lower seropositivity rate in camels bred for their meat or for transport activities, which are mostly males, could also be linked with the fact that males are often separated from the herd (the two sexes are only mixed during the reproduction activities) and have thus less contacts with other camels (i.e. females and calves).

Surprisingly, there was no observed difference between nomadic and sedentary herds in the seropositivity rate or virus RNA positive rate. Two hypotheses may explain this pattern. Firstly, the sedentary lifestyle is found in animal production systems where animals live at high density in ‘commercial’ farms. In such situations the virus may be introduced more easily to the herd with animals being bought from other sources and the virus once introduced will amplify to infect most of the susceptible animals, since they are in close contact with each other. The virus appears to have a density dependent transmission pattern. In contrast to this, nomads are long-distance travellers who connect different regions. Consequently they have multiple opportunities to come into contact with other camel populations during their travels, or through indirect contacts with water points and thus increasing the probability of encountering animals shedding MERS-CoV. In support to these interpretations, the lowest seroprevalence was found for the mixed lifestyle which is associated with medium herd sizes and relatively small range movements.

Our survey was limited to a narrow period in time, February–March 2015, and does not provide insights into seasonal variation in epidemiological dynamics. However, the synchronicity of the study across the different study sites is important because virus shedding may be related to seasonal and breeding cycles across these diverse geographical regions. By keeping this variable within relatively narrow bounds, we are able to meaningfully analyse the other parameters that impact on virus transmission dynamics within dromedary populations. Further studies should follow camel populations through the year to define seasonal variation in virus activity.

The results of our study are coherent with risk factors highlighted by Alraddadi and colleagues for human illness in Saudi Arabia [40]. They show, using a case–control design for exploring environmental exposures among primary case-patients from March to November 2014, that direct exposure to dromedary camels and particularly milking camels was significantly associated to MERS-CoV illness. These results consolidate the risk factors identified in our study on the camel females and milking activities [40]. Our results also give rise to a number of research questions to be followed up in future studies on MERS-CoV transmission dynamics in camel herds. In particular, the role played by young camels and the relationship with the mother need to be investigated more thoroughly.

Longitudinal investigations should also be undertaken in naturally-infected camels in different production systems and different age groups. Such investigations could provide valuable information on virus shedding in excretions (nasal, faecal, milk and urine) and on whether the virus is present in meat. It could also give insights into the dynamics of immunity in camels and reinfection mechanisms. Joint research on risk factors for transmission of MERS-CoV between camels, from camels to humans and from humans to camels should also be encouraged.

Genetic and phenotypic characterisation of MERS-CoV from Burkina Faso, Ethiopia and Morocco is required to understand how MERS-CoV in camels evolves within the continent, particularly with regard to capacity for inter-species transmission to humans.

Our study is one of the few studies that have so far addressed the influence of dromedary lifestyle on MERS-CoV infection as assessed by rate of seropositivity. While the study by Deem et al. (2015) in Kenya did not identify the factors associated with variation of seropositivity among farms [41], our study, which included different countries with a larger geographical range and included a larger number of farms with defined herd size, herd lifestyles and camel functions allowed us to explore associations of these factors with seropositivity. Such data contribute to understanding factors contributing to MERS-CoV infection in camels, which in turn might also have an effect on zoonotic infection. While we carried out our study in different parts of Africa, due to the fact that we have encompassed diverse geographical and ecological variables, our study findings may well be relevant in regions such as Saudi Arabia where zoonotic MERS remains a recurrent threat. Furthermore, it is not clear that transmission of MERS-CoV to humans is absent in Africa. A recent study has reported evidence of humans with MERS-CoV seropositivity in Kenya [22]. Further studies are needed to assess whether or not zoonotic MERS-CoV transmission occurs in Africa and our epidemiological data provide identification of situations of highest risk. Better understanding of the risk factors and virus transmission dynamics of MERS-CoV within camels is important in responding to the global health threat posed by MERS-CoV.
